# Notes and News

**Published:** 1888-09-15

**Authors:** 


### NOTES AND NEWS.

COLLECTED AND COMMUNICATED.

IN anticipation of the opening of the new session at the various medical schools, we publish this week a special "students' number." We have not thought it desirable to fill our columns with abridgments of the "calendars" and "regulations" of the various schools and degree-conferring bodies, but rather to offer counsels and suggestions which may be helpful alike to students, parents, and teachers. Those who desire detailed information concerning medical schools may readily obtain it by personal or written application. In London almost every general hospital has its own medical school. The medical schools of the provinces are in association with the Universities of Oxford, Cambridge, Durham, and the Victoria University at Manchester, as well as with the infirmaries of Leeds, Liverpool, Birmingham, and elsewhere. In Scotland there are four universities which confer medical degrees, and two other licensing bodies. In order to obtain complete information concerning any or all of these institutions, all that is needed is to write to the secretary in each case for a copy of the regulations relating to medical study. It is well, before finally deciding what course to pursue, that the student should have some knowledge of the relative advantages and disadvantages of the different schools.

DR. ROBERT LIVEING has been appointed consulting physician for skin diseases at the Middlesex Hospital.

IT is expected that the winter session of The Hospitals Association will be opened by a popular paper on "The Position and Prospects of the National Pension Fund for Nurses," by the Honorary Manager, Mr. Edward J. Clifford.

THE National Pension Fund for Nurses has advanced by leaps and bounds during the last two months. The issue of the new prospectus has produced results in the way of proposals beyond the most sanguine anticipations.

THE approach of the "railway accident season" reminds us that London is still to a large extent destitute of "ambulance accommodation." The recent correspondence in the daily papers, which had its origin in these columns, brought out the fact that at St. Mary's there is an efficient ambulance, and that there is no difficulty in finding horses for it when it is required for use. But in a city of railways, tramway cars, omnibuses, and busy thoroughfares like London a score ambulances at twenty different points would not be sufficient for the real necessities of the case. It may be hoped that another winter will not be allowed to pass without at least a good commencement being made to supply the lack. The Hospitals Association must see that definite steps are taken at the very beginning of the winter. Those who let the grass grow under their feet do not commend themselves to a practical generation.

THE whole country has been startled by another of thos terrible and inexplicable murders which have for some months been alarming the East-end of London. There is no necessity to report the facts, which have been given in more than sufficient detail by the daily papers. We add our protest to the cry that the police must not relax their vigilance till the murderer or murderers are securely in their charge. Whether there be a madman at large, or some human monster still more terrible than a homicidal maniac, the world demands that he shall be promptly laid hands on. All right-minded men must rejoice that the parish of St. Jude's, Whitechapel, is bestirring itself in a practical and intelligent way in the interests alike of public morality and safety. It may be hoped that vigilance associations such as that in St. Jude's parish will be multiplied by the hundred.

BY the kind permission of the proprietors of Madame Tussaud's Exhibition, Marylebone Road, twenty-five children from the Metropolitan and City Police Orphanage, Twickenham, accompanied by Mrs. Williams, the matron, and Miss Dudley, assistant mistress, visited the exhibition on Saturday last. Superintendent Draper, of the D division, arranged for Inspector Parsons to meet the party at the Baker Street railway station, and he conducted the children through the building. Afterwards they were provided with tea, and then left for home. These children were not only orphans, but were friendless, and this treat was arranged while the other children of the institution were keeping holiday with friends or relatives.

THE committees and secretaries of hospitals in London will have their hands decidedly full during the coming winter. The recent facts brought out by the Hospital Sunday Fund have stimulated public curiosity on the question of hospital expenditure. The "New Inquisition" has fairly commenced. It is certain that the public are eager for the continuance of its operations. Waste and extravagance are shameful in themselves; and when the waste is of funds supplied for charitable purposes, and often by those who can only spare a little help by severe self-denial, the waste becomes intolerable. All classes of officials must bestir themselves if they wish to avoid being overwhelmed in the avalanche of angry criticism which is threatening on all sides.

THE *Manchester Examiner* states that "At the Manchester Infirmary Board meeting recently Mr. O'Hanlon gave some particulars with regard to overcrowding in this city which almost surpass belief. In inquiring into the antecedents of a case of typhus fever, he found that a man, his wife, and ten children, varying in age from one to seventeen years, had all been sleeping in one room, the dimensions of which were 12 ft. by 9 ft. Each of them had about 81 cubic feet of air. The allowance at Monsall Hospital is 2,000 cubic feet for each person, so that it is easy to surmise how vitiated the atmosphere must be in which these poor creature live. The question of morals, all-important as it is, seems almost to shrink into secondary importance in view of the pressing problem of how these people breathe. Mr. O'Hanlon suggests that these facts may furnish us with an explanation of the high mortality rate of Manchester. But it may well be doubted whether such conditions as these are common anywhere, or whether they are more common in Manchester than in other large cities. Our bad pre-eminence in the matter of the death rate must, we fear, be explained by conditions of a more general character, and we look forward with great hopefulness to the improvements in the conditions of the Irwell as a means of bringing us to a level of equality with other large towns."

The following vacancies are announced this week, the amount of salary in each case being given after the name of the institution :—

**Resident Medical Officers.**

Belgrave Hospital for Children. House Surgeon and Assistant Secretary.—£30 per annum, with board, etc.
Cheltenham General Hospital. Resident Surgeon for the Branch Dispensary.—£180 per annum, with partly furnished house.
County Asylum, Shrewsbury. Junior Assistant Medical Officer. —£100 per annum, with board, etc.
East London Hospital for Children, Shadwell, E. Resident Clinical Assistant.—Board; no salary.
Fulham Union. Assistant Medical Superintendent.—£100 per annum, with board, etc.
General Infirmary, Hull. House Surgeon.—£105 per annum, with board, etc.
General Infirmary, Leeds. Resident Medical Officer and Pathologist —£100 per annum, with board, etc.
Salford Royal Hospital. House Surgeon.—£120 per annum, with board, etc
Salford Royal Hospital. Pendleton Branch Dispensary. District Surgeon.—£80 per annum, with board, etc.
West London Hospital, Hammersmith Road. House Surgeon.— Board; no salary.

**Non-resident Medical Officers.**

Manchester Royal Infirmary, Dispensary, and Lunatic Asylum. Honorary Assistant Physician.
University of Aberdeen. Chair of Chemistry.

**In-door Porter.**

Great Northern Central Hospital, Holloway. In-door Porter. £25 to £28 per annum.

---

The *New Zealand Times* says: "The establishment of Mr. J. E. Hayes, Lambton Quay, was yesterday the scene of an exhibition which we think decidedly unique in the history of engineering concerns. We refer to the trial of Spratt's Patent 'improved preparation of food for animals, game, and poultry,' etc., the subject-matter of which was compounded into dough, stamped into square biscuits, baked in a gas oven and served up hot, in the presence of gentlemen who evinced great interest in the proceedings, and who expressed the opinion when the finished article came into view, that it looked quite tempting enough for higher game than specified in the title. The patent food is compounded of wheat—flour, oatmeal, lentil-flour, flesh reduced to pulp, dates, sulphur, phosphate of lime, carbonate of iron, and any suitable spice—as essence of peppermint for instance—together with as much beetroot as will amount to about one-tenth in weight of all the other constituents. The food is evidently designed to provide in a handy form all the farinaceous and mineral elements necessary for the animal economy, and we have no doubt would prove highly beneficial to the health of domestic animals provided with it."

---

The Rev. Dr. Bruce, Chairman of the Committee of the Newcastle-on-Tyne Infirmary, at its weekly meeting, recorded a sad week's serious casualties at that institution. He said : "On Thursday a boy was admitted who had fallen from the roof of a house, suffering from a ruptured kidney and broken ribs. Injuries to the kidneys, it need not be mentioned, are very serious. On Saturday a man, whose thigh had been fractured by a fall of copper ore, came in. On the same day a boy who had fallen over the cliff at Tynemouth, and had fractured his skull, was received. A man was received the same day who had fallen in the streets and broken his leg. On Sunday a man suffering from a compound fracture of the skull, in consequence of a blow from a coal-rake, was brought in. The bone was driven right into the brain, and the operation of trephining was necessary. On Monday a man who slipped in the Central Station and broke his leg was received, on the same day a boy, who had been run over by the tubs in the pit, and had both his legs broken and otherwise injured, was brought in. The fracture of his right leg was a compound fracture. On Tuesday a boy suffering from concussion of the brain, in consequence of a fall from a cart, was admitted. On the same day a woman with a broken leg, the consequence of a fall, was received ; a child also came in who had swallowed some sulphuric acid. This list, striking as it is, is but an average one. Besides, it records only the more severe accidents, not the slighter ones, and it is silent as to the medical cases and the general work of the Infirmary. All the persons mentioned in it survive, except the child who swallowed the oil of vitriol, and it is hoped they will do well."

---

The vacancy in the office of resident physician to Bethlem Royal Hospital for Lunatics has called forth an important letter from a correspondent of the *City Press*, signing himself "Neurologist." It is obviously impossible that one resident physician, even with the aid of an assistant and two clinical clerks, can properly conduct the treatment of three hundred cases of insanity, attend to a heavy official correspondence, and the claims of a large consulting practice. At present the medical staff is organised on the county pauper lunatic asylum model, which is scarcely suitable for a place richly endowed, receiving a number of curable cases, and in close proximity to the chief medical schools. "Neurologist" suggests that Bethlem Hospital should have four physicians in charge of eighty beds each, and that two of these physicians should visit its wards daily, that it should also have two assistant physicians in charge of an out-patient department, to which persons threatened by insanity or suffering from it in its incipient stages and milder forms, when it may so often be arrested or cured, or convalescent after an attack, might resort for advice and treatment. These and other important suggestions are worth consideration, and it is to be hoped the vacancy will not be hastily filled up.

---

"Marriage" and Mrs. Mona Caird having now become threadbare, the new topic of "How to Live Long" has become the football of the "silly season." On this question Mr. W. Withnall, writing to the *Daily News*, says: "Will you permit me to put a few questions to Mr. Arthur T. Barnard, relative to his effusion on 'How to Live Long'? 1. Are docility and gentleness the only attributes of the perfect man? 2. Are they the paramount and exclusive traits of the dominant, *i.e.*, the meat-eating races of the earth? 3. If he objects to kill animals for food on the ground of morality, does he, in order to be consistent, refuse to wear their skins on his feet, their wool on his back, and to use their bones and ivory for useful and ornamental purposes? 4. Would he deprive his cat of bread and milk because the structure of its teeth indicates that it is a carnivore? 5. Is he not aware that the lowest savages live upon wild fruits, berries, nuts, roots, etc.? 6. Does the stomach of the civilized man most resemble that of the tiger or the ox; and which of these two would get the best of it in a rough-and-tumble struggle for existence? 7. Does he consider milk, eggs, cheese, and butter vegetable food; and, if not, how can a man be a vegetarian and consume them? 8. If he consumes them, how does he arrive at the conclusion that his clean bill of health is due to vegetarianism? 9. May not the disease germs of which he speaks be present in milk as well as meat, and will not efficient cooking destroy them? 10. If the people who dwell in tropical climates find a vegetarian diet best suited to their requirements, and the inhabitants of arctic regions can only maintain life upon animal food, what should be the most desirable dietary for dwellers in the temperate zones? 11. If the fact that all the constituent parts of the human body are found in fruits, grains, and nuts prove that they are desirable food, why don't we eat sawdust, which likewise contains those elements? 12. Does not 'instinct lead mankind in different climates to select food in accordance with the varying conditions under which they live?' 13. How is it that I, a chronic dyspeptic, can digest and thrive upon mutton, fish, and game, whilst a vegetarian diet induces flatulence and indigestion, and impoverishes my whole system?"

